# Unlocking the Stratum Corneum Barrier to Skin Penetration for the Transdermal Delivery of Cyclovirobuxine D

**DOI:** 10.3390/pharmaceutics16121600

**Published:** 2024-12-16

**Authors:** Yun-Hao Ren, Feng-Yuan Song, Jing-Yu Zhao, Bing-Wen Liang, Li-Hua Peng

**Affiliations:** 1 College of Pharmaceutical Sciences, Zhejiang University, Hangzhou 310058, China; renyunhao@nwafu.edu.cn (Y.-H.R.); sfy1994@126.com (F.-Y.S.); 22360538@zju.edu.cn (J.-Y.Z.); 2 Jinhua Institute of Zhejiang University, Jinhua 321299, China; 3 Hospital of Nanjing Junxie, Nanjing 210002, China; bingwen_liang@sina.com

**Keywords:** cyclovirobuxine D, transdermal delivery, skin penetration enhancement, niosomes, chemical penetration enhancers

## Abstract

**Background/Objectives:** Cyclovirobuxine D, a natural compound derived from the medicinal plant Buxus sinica, demonstrates a diverse array of therapeutic benefits, encompassing anti-arrhythmic properties, blood pressure regulation, neuronal protection, and anti-ischemic activity. However, its limited solubility hinders the bioavailability of current oral and injectable formulations, causing considerable adverse reactions and toxicity. **Methods:** In this investigation, we embarked on an unprecedented exploration of the skin penetration potential of cyclovirobuxine D utilizing chemical penetration enhancers and niosomes as innovative strategies to enhance its dermal absorption. These strategies were rigorously tested and optimized. **Results:** Among the tested chemical penetration enhancers, azone emerged as the most potent, achieving a 4.55-fold increase in skin penetration compared to the untreated group. Additionally, when encapsulated within niosomes, primarily composed of Span60 and cholesterol, the skin penetration of cyclovirobuxine D was notably enhanced by 1.50-fold. Furthermore, when both cyclovirobuxine D and azone were co-encapsulated within the niosomes, the skin penetration of cyclovirobuxine D was remarkably elevated by 8.10-fold compared to the solvent-dispersed group. This enhancement was corroborated through rigorous in vitro and in vivo experiments. Notably, the combination of other chemical penetration enhancers with niosome encapsulation also exhibited synergistic effects in enhancing the skin penetration of cyclovirobuxine D. **Conclusions:** These findings provide a compelling rationale for the administration of cyclovirobuxine D via skin-mediated transdermal delivery, offering superior safety, efficacy, and convenience. The innovative combination of niosomes and chemical penetration enhancers represents a novel system for the transdermal delivery of cyclovirobuxine D, holding immense promise for clinical applications in the treatment of brain, neuronal, and cardiovascular disorders.

## 1. Introduction

**Figure 1:**
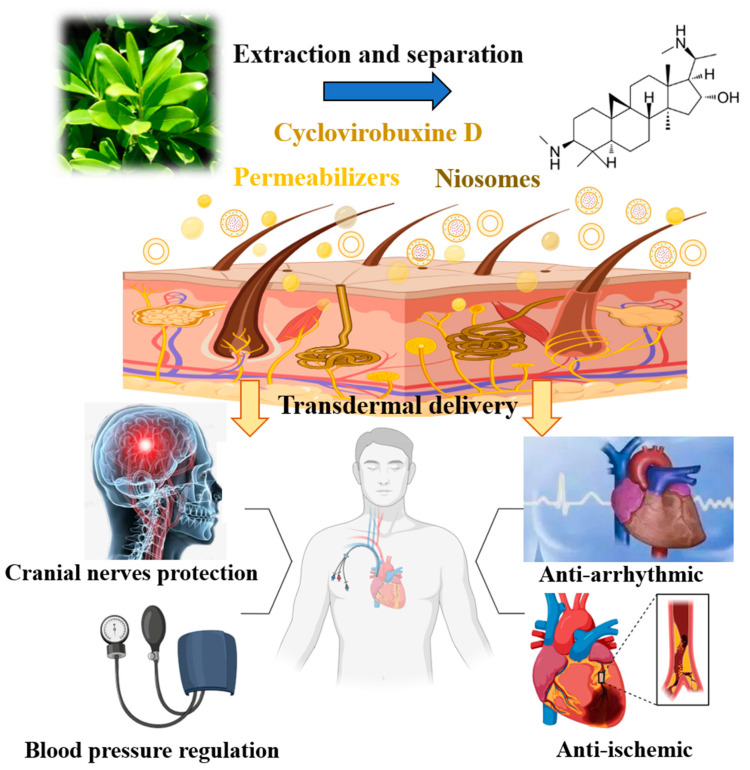
Extraction and separation, capability for the transdermal delivery and disease treatment of cyclovirobuxine D.

Cardiovascular and cerebrovascular diseases represent prevalent conditions among the elderly in our aging society and are characterized by their high incidence, severity, and mortality rates. The repertoire of safe therapeutic agents within this domain remains woefully inadequate. Notably, cyclovirobuxine D, an active natural compound derived from the medicinal plant *Buxus sinica*, holds immense clinical potential in treating these diseases. It demonstrates a broad spectrum of therapeutic activities, including addressing cerebrovascular disorders and neuropathies [1], but also exhibits remarkable therapeutic versatility in addressing chronic ailments such as cardiovascular and cerebrovascular diseases [2], correcting arrhythmias [3], modulating blood pressure, and treating ischemic heart failure [4], among others (Figure 1). This compound achieves these benefits by enhancing intracellular Ca^2+^ utilization, which alleviated cardiac dysfunction in rats with congestive heart failure [5], and significantly inhibited the progression of heart failure [6]. Conversely, it mitigated the influx of Ca^2+^ through L-type Ca^2+^ channels, modulated iron metabolism, and alleviated cardiac iron toxicity induced by sepsis [7]. Furthermore, cyclovirobuxine D demonstrated potential in treating diabetic cardiomyopathy (DCM) by inhibiting oxidative stress through the activation of the Nrf2 signaling pathway [4]. By concurrently activating Nrf2 and SIRT3, it improved aldosterone regulation [8] and protected against cardiomyopathy by mitigating oxidative damage and maintaining mitochondrial biogenesis [7]. Despite its promising therapeutic potential, the unique physicochemical properties of cyclovirobuxine D pose significant challenges. Its low solubility in water, chloroform, methanol, ethanol, and acetone (approximately 0.06 mg/mL in water) [9,10], combined with an oil–water partition coefficient of 2.4 [10], result in poor bioavailability. Traditional oral and injection routes exacerbate these issues due to the prominent first-pass effect and notable toxicity to the liver, kidney, and reproductive system. Moreover, the lack of innovative delivery systems for cyclovirobuxine D further restricts its clinical application. These limitations significantly hinder the utilization of cyclovirobuxine D in medical practices.

The transdermal drug-delivery system (TDDS) represents an advanced method of administering medications through the skin to achieve either local or systemic therapeutic objectives. It ranks as the third most prevalent delivery system, preceded only by oral and injectable routes. Consequently, in recent years, the increasing number of painless and non-invasive TDDS has garnered significant attention for the management of chronic conditions such as cardiovascular and cerebrovascular diseases, neuropathy, and other ailments. Skin-mediated TDDS minimizes the risk of overdose associated with oral or injectable administration [11,12]. These systems enable drugs to be absorbed through the skin at a controlled rate via the capillaries, facilitating their entry into systemic circulation, thereby achieving systemic therapeutic goals [13,14]. By effectively bypassing first-pass liver metabolism and the rapid degradation of drugs in the gastrointestinal tract, TDDS enhances drug bioavailability and improves patient compliance. Furthermore, it supports sustained drug release [15,16]. Given these numerous advantages, we have chosen TDDS as the delivery method for cyclovirobuxine D.

However, the dense stratum corneum of the skin presents a formidable barrier to the penetration of external substances, particularly those with low solubility, often necessitating additional penetration enhancement techniques. Currently, research efforts are intensely focused on enhancing penetration through the utilization of physical, chemical, and pharmaceutical strategies. Among these, chemical penetration enhancers and nanotechnology stand out as two groundbreaking approaches for the transdermal delivery of hydrophobic compounds and biomolecules [17]. Nanotechnology-based drug-delivery systems have played a pivotal in overcoming the limitations associated with traditional dosage forms [18]. Niosomes, which have the capability to carry a diverse range of drug molecules, have emerged as promising carriers for drug delivery. In this study, we embarked on an in-depth investigation of the transdermal properties of cyclovirobuxine D solution by examining various ratios of receiving liquids. Subsequently, we screened a multitude of chemical penetration enhancers listed in the pharmacopoeia to identify those with exceptional skin penetration-enhancing effects on cyclovirobuxine D. Furthermore, we formulated niosomes containing cyclovirobuxine D to assess their penetration-enhancing capabilities. Additionally, we incorporated chemical penetration enhancers into the niosomes and evaluated their combined effects on the skin penetration of cyclovirobuxine D through both in vitro and in vivo rat experiments. The findings of this study provide novel insights into the transdermal delivery of cyclovirobuxine D. In conclusion, the combination of azone with niosomes emerges as an innovative and efficient formulation for the safe and convenient transdermal delivery of cyclovirobuxine D.

## 2. Materials and Methods

### 2.1. Materials

Cyclovirobuxine D (Shanghai Yuanye biotechnology Co., Ltd., Shanghai, China); retinol and glabridin (Shanghai Liding biotechnology, Shanghai, China); azone, borneol, oleic acid, and ammonium acetate (Nanjing Chemical Reagent, Nanjing, China); formic acid (Switzerland Wokai, Geneva, Switzerland); anhydrous ethanol (Shanghai Titan Technology, Shanghai, China); phosphotungstic acid (Shanghai Meryer, Shanghai, China); acetonitrile, PEG400, NaCl, Span60, ethanol, and cholesterol (Germany Merck, Darmstadt, Germany); CCK-8 kit (Hangzhou Mingte, Hangzhou, China); Schwann cells (RSC96), human epidermal keratinocytes (HaCaT) and human skin fibroblasts (BJ) were originated from early laboratory cultures.

### 2.2. Instruments

The following instruments were used: an automatic transdermal diffusion instrument (LOGAN); ultra-high performance liquid chromatography (UHPLC) (Japan Shimadzu, Kyoto, Japan); triple quadrupole mass spectrometer (AB Science); Agilent polaris 3C_18_-A (100 × 2.0 mm) chromatography column (US Agilent, Santa Clara, CA, USA); constant temperature water bath (Shanghai Yiheng, Shanghai, China); centrifuge (Shanghai Luxiangyi, Shanghai, China); electronic analytical balance (Odolis, Cardiff, China); ultrapure water machine (Hong Kong Likang Heal Force, Hong Kong, China); rotary evaporator (Japan Yamato); zetasizer nano ZS Zen3600 (Malvern Instruments Inc., Malvern, UK); and a transmission electron microscopy (TEM) (JEM-1200EX, JEOL Ltd., Tokyo, Japan).

### 2.3. Animals

Sprague–Dawley (SD) rats (female, 4-week-old, 100 ± 20 g) and New Zealand white rabbits (1.5 ± 0.5 kg) were supplied by Shanghai SLAC laboratory animal company (Hangzhou, China). All animal tests were in accordance with the experimental animal welfare and ethics committee of Zhejiang University and were approved by all animal care and experimental procedures. The ethics committee’s approval number is “ZJU20170733”.

### 2.4. Preparation of Rat Skin

The rats were anesthetized by the intraperitoneal injection of pentobarbital sodium (3%, 0.2 mL/100 g for rats). The dorsal hair of the rats was meticulously shaved while ensuring the integrity of the stratum corneum (SC). Subsequently, the subcutaneous fat was carefully dissected from the skin and any connecting tissue was removed. The resultant tissue was rinsed with saline, placed in rings, and stored at −20 °C for future use.

### 2.5. Skin Penetration Tests In Vitro

The skins of the rats were utilized for a penetration test with Franz diffusion cells in vitro. The skin was cut to an appropriate size and securely mounted onto the receptor compartment of the diffusion cell, with the appropriate receiving liquid selected for the experiment, and the volume of the receiving liquid in each sample is 17 mL. The cyclovirobuxine D was applied to the donor cells. The receptor was maintained at 300 r/min stirring with a magnetic bar and 32 ± 0.5 °C during the experiments. Each experiment was conducted in triplicate to ensure accuracy. At predetermined intervals, 1 mL of the receiving liquid was withdrawn and replaced with an equal volume of fresh receiving liquid. The withdrawn samples were filtered through a 0.22 μm filter using a water system microporous membrane and prepared for liquid chromatograph–mass spectrometer (LC–MS) detection.

### 2.6. Skin Penetration Receiving Liquid

PEG400: ethanol: saline was selected as the receiving liquid in a volume ratio of 1:3:6 (pH 6.7), 2:2:6 (pH 6.2), and 2.5:2.5:5 (pH 5.8), respectively. The cyclovirobuxine D solution 2.5 mg (100 μL, 25 mg/mL) was applied to the donor cells. The receiving liquid was kept at 300 r/min stirring with a magnetic bar. Then, 32 ± 0.5 °C. 1 mL of the fresh receiving liquid was withdrawn for LC–MS detection.

### 2.7. Chemical Penetration Enhancers

A volume ratio of ethanol: saline = 3:7 was selected as the receiving liquid (pH 7.2). The cyclovirobuxine D solution 1.5 mg (100 μL, 15 mg/mL) and 0.75 mg (50 μL, 15 mg/mL) of the solution with 5 chemical penetration enhancers were applied to the donor cells. The solvent used in the experiment, after mixing, was absolute ethanol at a concentration of 10 mg/mL for the cyclovirobuxine D and 5 mg/mL for the chemical enhancers. The chemical penetration enhancers included retinol, glabridin, borneol, azone, and oleic acid. The receiving liquid was maintained at 300 r/min stirring with a magnetic bar, and 32 ± 0.5 °C. 1 mL of the fresh receiving liquid was replaced and prepared for LC–MS detection.

### 2.8. Preparation of Cyclovirobuxine D-Loaded Niosomes

The preparation method for the cyclovirobuxine D niosomes was the thin-film dispersion method. Specifically, 1.5 g Span60, 1.5 g cholesterol, and 0.2 g cyclovirobuxine D were dissolved in 25 mL of ethanol and subjected to reduced pressure rotary evaporation at 40 °C until a thin film formed at the bottom of a round bottom flask. Following an overnight stay at room temperature, 20 mL of a PBS buffer solution (pH 7.5), preheated to 55 °C, was introduced to hydrate the film. Under a consistent temperature of 55 °C, magnetic stirring was carried out for 1 h, followed by an overnight resting period in a refrigerator at 4 °C. On the following day, the mixture underwent low-temperature ultrasound treatment at 100 W for 2 h. After filtration through a 0.45 μm filter membrane, cyclovirobuxine D niosomes (Cy-Nio) were obtained and stored in a refrigerator at 4 °C. The preparation process for the blank niosomes mirrored that of the Cy-Nio, with the exception of the omission of cyclovirobuxine D. The total input of cyclovirobuxine D amounted to 1 mg (100 μL, 10 mg/mL).

The encapsulation efficiency of the Cy-Nio was determined using low-speed centrifugation and membrane filtration methods. Precisely 2 mL of a Cy-Nio suspension was accurately measured and centrifuged at 3000 rpm for 10 min. Due to the small particle size of Cy-Nio, it is difficult to precipitate due to its suspended nature. Since Cy is insoluble in water and exists as microparticles, it can be separated and precipitated through centrifugation. Subsequently, a 0.45 μm microporous filter membrane was used to filter the Cy-Nio suspension, effectively removing any residual unencapsulated Cy. A 1 mL aliquot of the filtered suspension was accurately measured and transferred to a 10 mL volumetric flask. Methanol was added and the mixture was vortexed and thoroughly mixed. The emulsion was disrupted through sonication for 30 min and the volume was adjusted to the mark with further mixing. The sample was then injected under chromatographic conditions to determine the Cy content, allowing for the calculation of the Cy content within the niosomes (m_1_). The total dosage of Cy (m_2_) was also calculated, and the encapsulation efficiency of Cy-Nio was derived using the following formula [19]:


$$Encapsulation\;rate\left(\%\right)=\frac{m1}{m2}\times 100\%$$

### 2.9. Particle Size and Zeta Potential

The mean particle size of the Cy-Nio was determined using photon correlation spectroscopy with the Zetasizer Nano ZS Zen3600 at temperatures of 25 °C and 173 °C. These measurements were obtained using a helium-neon laser. Zeta potential (ZP) measurements were carried out at 25 °C in folded capillary cells using the same instrument. The ZP values were obtained from the electrophoretic mobility using the Smoluchowski equation. All measurements were conducted in triplicates to ensure accuracy.

### 2.10. Morphological Analysis by TEM

The morphology of the niosomes was observed using transmission electron microscopy (TEM). The sample was prepared by placing a drop of the formulation, which had been diluted 50-fold with double-distilled water, onto a 400-mesh copper grid coated with a carbon film. This was followed by negative staining with 1% phosphotungstic acid to enhance visibility.

### 2.11. Stability Evaluation of Cyclovirobuxine D

A total of 1 mg/mL of the cyclovirobuxine D solution and Cy-Nio, respectively, were prepared; the cyclovirobuxine D was dissolved in absolute ethanol and the Cy-Nio was dissolved in PBS (pH 7.5). The photostability (wrapped in tin foil and exposed to indoor light for 1, 5, 10, and 15 d), storage stability (refrigerated at 4 °C and stored at room temperature for 1, 5, 10, and 15 d), thermal stability (heated at 50 °C for 1 h, heated at 37 °C for 1 h, and left at room temperature for 1 h), and acid-base stability (adjusted to pH 3, 7, and 11) were evaluated and the content of the cyclovirobuxine D and particle size of the niosomes in the solution were determined. The acidity (or alkalinity) of the Cy-Nio was determined using pH test strips.

### 2.12. Optimization of Skin Chemical Penetration Enhancers for Niosomes

A volume ratio of ethanol: saline = 3:7 was selected as the receiving liquid. The solution ratio screened with better skin permeation effect was used for percutaneous skin permeation enhancement of Cy-Nio and 0.75 mg (50 μL, 15 mg/mL) retinol, glabridin, borneol, azone, and oleic acid as chemical penetration enhancers, respectively. The total amount of cyclovirobuxine D added was obtained by multiplying m_2_ by the encapsulation rate described in Section 2.8. Other methods are the same as those described in Section 2.5.

### 2.13. Skin Penetration Tests In Vivo and Pharmacokinetic of Cyclovirobuxine D

A total of 10 mg/mL of the cyclovirobuxine D solution and Cy-Nio were prepared and 9 Sprague–Dawley (SD) rats (female, 4-week-old, 100 ± 20 g) were selected and randomly divided into the following 3 groups: cyclovirobuxine D solution group by gavage 3 mg (300 μL, 10 mg/mL); cyclovirobuxine D solution applied to abdominal skin group 3 mg (300 μL, 10 mg/mL); and Cy-Nio applied to abdominal skin group 3 mg (300 μL, 10 mg/mL). Each rat was given 3 mg of cyclovirobuxine D. At time points 2, 4, 8, 12, and 24 h after administration, 200 μL/time of rat blood was collected using the tail vein method to plot the pharmacokinetic curve of cyclovirobuxine D. After 24 h of treatment, the rats were euthanized and the skin permeability of cyclovirobuxine D was measured by taking blood samples as well as heart, liver, spleen, lung, left kidney, right kidney, large intestine, small intestine, stomach, brain, and feces samples.

### 2.14. Establishment of LC–MS Detection Method for Cyclovirobuxine D Analysis

The LC–MS detection conditions were as follows.

The LC chromatographic column was an Agilent Polaris 3C_18_-A (100 × 2.0 mm); the mobile phase A was 0.1% formic acid water containing 10 mM ammonium acetate; mobile phase B was acetonitrile with a flow rate of: 0.35 mL/min and an injection volume of 5 μL. The gradient elution procedure is shown in Table 1.

The MS was conducted using the multiple reaction monitoring (MRM) positive ion mode with, as follows: a scanning time of 7.5 min; spray gas of 55 psi; auxiliary heating gas of 55 psi; curtain gas of 35 psi; ion source temperature of 550 °C; ion source voltage of 5500 V; and collision gas of 8 psi. The MRM ion pair parameters are shown in Table 2. Among these, multiple reaction monitoring (MRM) is a mass spectrometry technique based on known or assumed reaction ion information that selectively selects data for mass spectrometry signal acquisition, records signals of compliant ions, removes interference from non-compliant ion signals, and obtains quantitative mass spectrometry information through the statistical analysis of the data.

### 2.15. Cell Proliferation Assessment

The cytotoxicity of micelles was examined with a CCK-8 kit in vitro. Briefly, cells (5000 cells/well) were seeded on a 96-well plate and cultivated for 24 h. Subsequently, the cells were incubated with various concentrations of cyclovirobuxine D, Cy-Nio, azone, borneol, oleic acid, retinol, or glabridin for 24 h. After this treatment period, the original culture medium was replaced with a DMEM medium containing 10% CCK-8 reagent (without fetal bovine serum, FBS) and incubated for another 1 h. The optical density (OD) values were then measured using an infinite F50 absorbance enzyme-linked immunosorbent assay reader at a wavelength of 450 nm. Cell viability was calculated using the following formula. A represents the OD value of cells treated with the chemical compound (control) and B represents the OD value of blank control. Each experiment was conducted in triplicate to ensure accuracy, as follows:


$$Cellviability=\frac{\left(A-B\right)}{B}\times 100\%$$

### 2.16. Skin Irritation Test

Eight New Zealand white rabbits were randomly assigned to 4 groups with 2 rabbits in each group, as follows: an untreated intact skin group; an intact skin + Cy-Nio (containing azone) group; scratched skin; and a scratched skin + Cy-Nio (containing azone) group. Prior to administration, a skin integrity examination was conducted; any individuals with pre-existing skin damage were excluded from the intact skin group. Twenty-four hours before administration, a shaver was used to remove the hair on both sides of the back of the rabbits, within a range of 3 cm × 3 cm. The skin was then scratched using a needle to abrade the epidermal layer (confirmed by the presence of bleeding). Cy-Nio was directly applied to the shaved skin, followed by the placement of two layers of gauze (2.5 cm × 2.5 cm) secured with non-irritating tape and a bandage for 4 h. This process was repeated twice daily at the same site for 2 d; the results were observed on day 3. The scoring criteria for skin irritation are outlined in Table 3.

### 2.17. Data Calculation and Analysis

According to the following equation, A represents the effective diffusion area, *V* is the sampling volume, *V’* is the total volume of the receiving liquid, and *Cn* and *Ci* are the drug concentrations at the *n*-th and *i*-th sampling points, respectively. The cumulative skin permeability *Q* (μg/mL) of the drug per unit area can be calculated. To assess the penetration-promoting ability of chemical penetration enhancers, the enhancement ratio (ER) is defined as the ratio of the skin penetration of cyclovirobuxine D with a specific enhancer to the skin penetration of cyclovirobuxine D without any enhancer, as follows:


$$Q\left(\mu g/{cm}^{2}\right)=(VCn+V'\sum_{i=1}^{n-1}Ci)/A$$

All values are presented as mean ± SEM. Statistical significance was determined using Student’s t-test. For multiple comparisons, a two-way analysis of variance (ANOVA) with Tukey’s multiple comparisons test was conducted. Differences were considered statistically significant at *p* < 0.05. * *p* < 0.05, ** *p* < 0.01, *** *p* < 0.001.

## 3. Results

### 3.1. Establishment of LC–MS Detection Standard Curve for Cyclovirobuxine D

During the process of skin penetration, the selection of an appropriate penetration receiving liquid or chemical enhancer necessitates a comparison of the compound’s penetration levels under different treatment conditions. Consequently, the initial step involves choosing a suitable method for detecting its concentration. However, due to the ultraviolet absorption wavelength of cyclovirobuxine D being below 210 nm, an ultraviolet edge effect arises, rendering it challenging to detect using an HPLC–UV detector due to a poor baseline. This study also attempted HPLC–ELSD detection but found that the detection limit was only at the microgram level, specifically, approximately 10 μg/mL [20], which was inadequate to achieve the nanogram level, potentially resulting in inaccuracies. Therefore, LC–MS was chosen to detect the concentration of cyclovirobuxine D in the receiving liquid following skin penetration.

**Figure 2:**
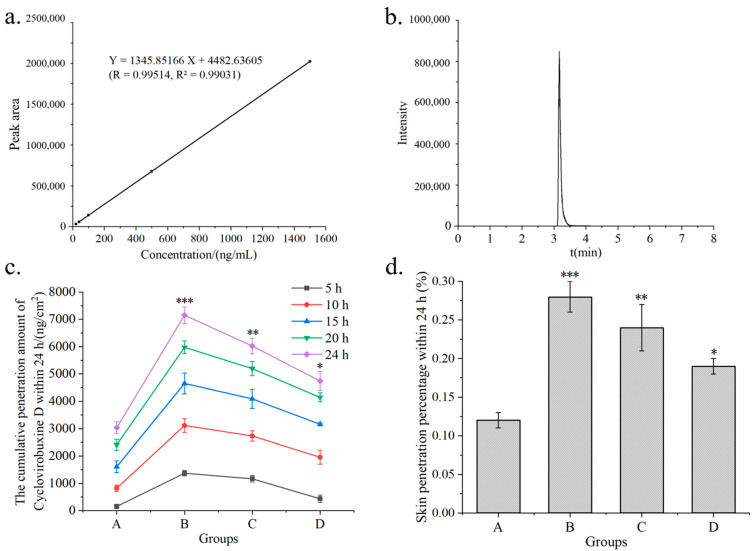
(**a**) standard curves obtained from different concentrations of cyclovirobuxine D samples by LC–MS; (**b**) peak time of cyclovirobuxine D samples under liquid quality conditions by LC–MS; (**c**) cumulative skin penetration and (**d**) cumulative skin penetration percentage (%) of cyclovirobuxine D under different receiving liquids A, B, C, and D. Among these, PEG400: ethanol: saline was selected as the receiving liquid, with volume ratios of 1:3:6, 2:2:6, and 2.5:2.5:5, respectively, represented by B, C, and D, where A is saline as the receiving liquid. * *p* < 0.05, ** *p* < 0.01, and *** *p* < 0.001.

Therefore, the sample dilution procedure was conducted as follows. First, 10 μL and 20 μL of a 2 μg/mL cyclovirobuxine D standard solution were taken and mixed with 990 μL and 980 μL of acetonitrile, respectively. Additionally, 10 μL aliquots of 10 μg/mL, 50 μg/mL, and 150 μg/mL cyclovirobuxine D standard solutions were each diluted with 990 μL of acetonitrile to prepare solutions of 20 ng/mL, 40 ng/mL, 100 ng/mL, 500 ng/mL, and 1500 ng/mL, respectively. The mixtures were vortexed for 30 s and then transferred to liquid chromatography–mass spectrometry (LC–MS) injection vials for analysis. The peak time for cyclovirobuxine D detected by LC–MS was 3.16 min (Figure 2b). After calculating the concentration and peak area of cyclovirobuxine D, the standard curve equation was derived as Y = 1345.85166X + 4482.63605, with an R^2^ value of 0.99031, as illustrated in Figure 2a. This equation fulfills all necessary criteria and will be utilized for subsequent concentration calculations.

The results of the analysis indicated that it is reasonable to use LC–MS for quantitative analysis; thus, it was possible to screen for the chemical penetration enhancers and niosomes of cyclovirobuxine D.

### 3.2. Optimization of Skin Penetration Receiving Liquid

The permeability of a transdermal substance is influenced by various factors, including the composition and pH value of the receiving liquid, skin type, the temperature of the diffusion cell, the concentration, amount supplied, and dosage form of the substance [21]. Given the poor solubility of cyclovirobuxine D, which prevents the formation of a leaky groove effect under conventional receiving liquids, a screening of receiving liquids for percutaneous permeation of cyclovirobuxine D solutions was conducted. The solubility of cyclovirobuxine D in absolute ethanol was 18 mg/mL at room temperature and pressure, and the solubility in PEG400, ethanol, and saline solution with volume ratios of 1:3:6, 2:2:6, and 2.5:2.5:5 were 11, 10.5, and 12.5 mg/mL, respectively. The total input amount of cyclovirobuxine D was 2.5 mg (100 μL, 25 mg/mL). It was found that when the volume ratio of PEG400: ethanol:saline was 1:3:6, the highest skin penetration of cyclovirobuxine D was observed within 24 h, with a skin penetration percentage of 0.28%. This was significantly higher than the skin penetration of cyclovirobuxine D in physiological saline, which was 0.12% (Figure 2c,d). However, as the amount of PEG400 was increased, it was observed that the skin penetration of cyclovirobuxine D decreased. This may be attributed to the viscosity of PEG400. Furthermore, research indicates that an increase in PEG400 may also inhibit the biological transformation of prednisolone esters, resulting in decreased efficacy [22]. Although the optimal receiving liquid was found to be one with a volume ratio of PEG400: ethanol: saline = 1:3:6, its effect was not exceptional. Moreover, incorporating an excessive amount of chemical components into the receiving liquid lacks practicality. Therefore, for the subsequent phase of chemical permeation agent screening experiments, we opted to decrease the PEG400 content by 10% and replenish it with saline.

### 3.3. Screening of Chemical Penetration Enhancers

**Figure 3:**
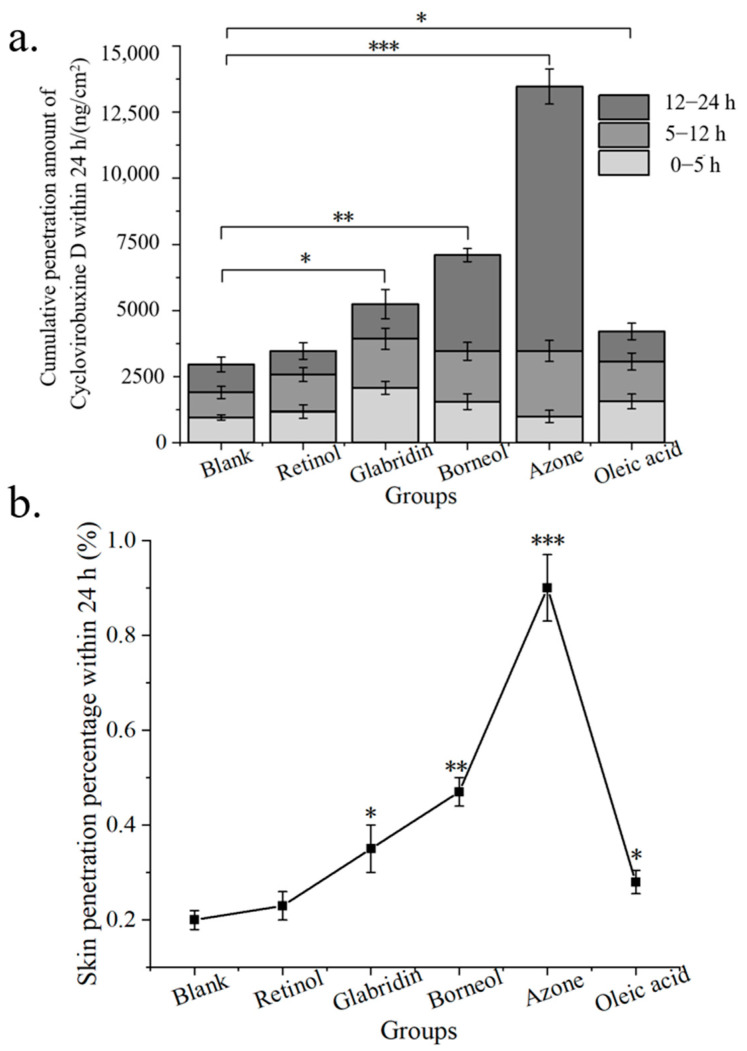
(**a**) cumulative skin penetration; and (**b**) skin penetration percentage (%) of cyclovirobuxine D under the condition of V (ethanol: saline) = 3:7 as the transdermal receiving liquid for different chemical penetration enhancers. * *p* < 0.05, ** *p* < 0.01, and *** *p* < 0.001.

Given the limited skin penetration of cyclovirobuxine D, a rigorous screening of chemical penetration enhancers for cyclovirobuxine D solutions was conducted. Initially, we assessed the skin penetration efficacy of 5 chemical penetration enhancers on cyclovirobuxine D, while eliminating the presence of PEG400. The total input of cyclovirobuxine D was 1.5 mg (100 μL, 15 mg/mL). A receiving liquid with a volume ratio of ethanol: saline of 3:7 was selected. The solubility of cyclovirobuxine D in a 3:7 volume ratio of ethanol and saline was 8.5 mg/mL at room temperature and pressure. We selected borneol, azone, retinol, glabridin, and oleic acid as the chemical penetration enhancers. Our findings revealed that the penetration percentage of cyclovirobuxine D within 24 h was 0.20% (Figure 3b), which surpassed the penetration effect observed with saline as the receiving liquid (Figure 2d). Among the enhancers, azone exhibited the most potent skin penetration effect on cyclovirobuxine D, achieving 4.55 times the penetration of the control group, followed by borneol (2.40 times), glabridin (1.77 times), oleic acid (1.42 times), and retinol (1.17 times) (Figure 3a,b). The permeability-related indicators of all tested penetration enhancers are shown in Table 4. Similarly, a study by Virani et al. demonstrated that all chemical penetration enhancers increased the permeability of oxcarbazepine (OXC) compared to the control [23]. Notably, azone’s minimal effect on keratin makes this non-invasive and efficient strategy a promising avenue for transdermal drug delivery and skin health management [24]. In conclusion, we selected azone, which demonstrated the strongest permeation-enhancing effect on cyclovirobuxine D, for further experimentation.

### 3.4. Optimization of Cyclovirobuxine D niosomes

**Figure 4:**
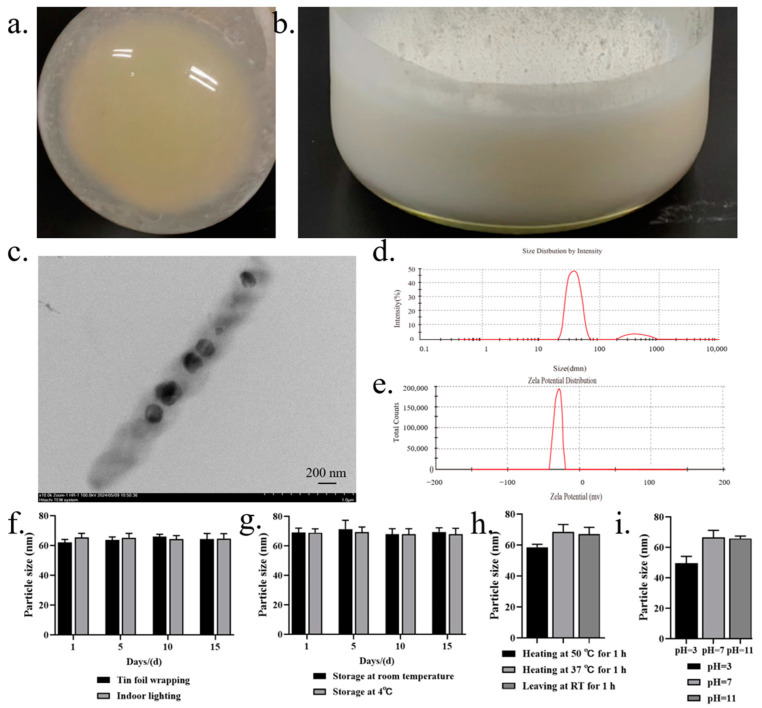
(**a**) pressure was reduced and evaporation was rotated at 40 °C until a yellow viscous solid film appeared at the bottom of the round bottomed flask; (**b**) after magnetic stirring for 1 h in a constant temperature environment of 55 °C, the white homogeneous liquid appears viscous; (**c**) Cy-Nio observed under a 15k transmission electron microscope; (**d**) particle size; and (**e**) potential distribution of niosomes. Evaluation of: (**f**) the photostability; (**g**) storage stability; (**h**) thermal stability; and (**i**) acid-base stability of Cy-Nio in the range of 1, 5, 10, and 15 d.

In the aforementioned system, cyclovirobuxine D and a chemical penetration enhancer coexisted solely in solution form, lacking the structure necessary to effectively constitute a formulation. Consequently, we contemplated the preparation of niosomes to encapsulate these components, with the aim of determining whether this encapsulation would further enhance the skin permeation of cyclovirobuxine D. The fabrication of lipid niosomes, analogous to liposomes, transfersomes, and exosomes, has been proven to augment transdermal drug delivery. Various established methods, such as the cold method, ethanol injection method, ether injection method, and thin-film hydration method, are employed for their preparation, each significantly influencing the characteristics of the niosomes. In this study, in the preparation of niosomes, the ideal ratio of Span 60 to cholesterol is 1:1. Although it is usually mentioned as a molar ratio, the relative molecular mass of span 60 is 430.62, while the relative molecular mass of cholesterol is 386.65; therefore, the difference between their relative molecular mass is not large, and the molar ratio can also be calculated simply as a weight-weight ratio. Therefore, a 1:1 weight-weight ratio was chosen for this study. We utilized the thin-film hydration method, which involved applying reduced pressure and rotary evaporation at 40 °C until a yellow, viscous solid film formed at the bottom of the round-bottomed flask (Figure 4a). Following magnetic stirring for one hour in a constant temperature environment of 55 °C, a white, homogeneous, viscous liquid emerged (Figure 4b). To gain further insights into the niosomes encapsulating cyclovirobuxine D, TEM studies were conducted. The TEM images revealed that the niosomes exhibited an almost spherical and homogeneous shape, albeit with slight irregularities at the particle periphery. The size was also uneven, with a range of 50–200 nm (Figure 4c). For the first time, the size of the cyclovirobuxine D-loaded niosomes was determined using a laser particle size analyzer, which demonstrated that niosomes were approximately 63 nm in diameter (Figure 4d); the measured result is also in the range of 50–200 nm. Furthermore, since TEM (transmission electron microscope) and particle sizers are distinct analytical instruments, it is expected that their results may differ. First, electron microscopy, exemplified by TEM, primarily focuses on observing the microstructure and composition within a sample, capturing images through the interaction between electrons and the sample. Conversely, particle size measuring instruments, like particle size analyzers, primarily measure macroscopic characteristics such as particle distribution and size. These instruments may utilize various principles, including laser diffraction, or sieving. Given the disparities in testing principles and methodologies, it is understandable that the results obtained by these two instruments may vary. Second, the operation of an electron microscope necessitates a vacuum environment to prevent electron scattering and interference. In contrast, particle size measuring instruments can operate at room temperature. Samples may exhibit differing properties in these different environments, leading to inconsistent test results. Lastly, particle size calculations typically rely on specific models or formulas, such as the Schiele formula (used for XRD data), which are based on certain assumptions and may thus contain a degree of error. On the other hand, electron microscopy measures particle size by directly observing the microstructure of the sample, providing a more intuitive result. However, in a TEM image, the size of niosomes can vary from their surface due to the inherent non-uniformity in the size of niosomes prepared from these capsules. Therefore, it is reasonable to consider the size of niosomes as a range spanning from 50 to 200 nm (Figure 4c and Figure S1); some portions of the niosomes measured by the particle size analyzer happened to be 63 nm in size in that time. Additionally, the zeta potential was approximately −25 mV (Figure 4e), within the acceptable range of −110 to 0 mV. Notably, the presence of cyclovirobuxine D molecules did not significantly alter the particle size or zeta potential of the niosomes.

**Figure 5:**
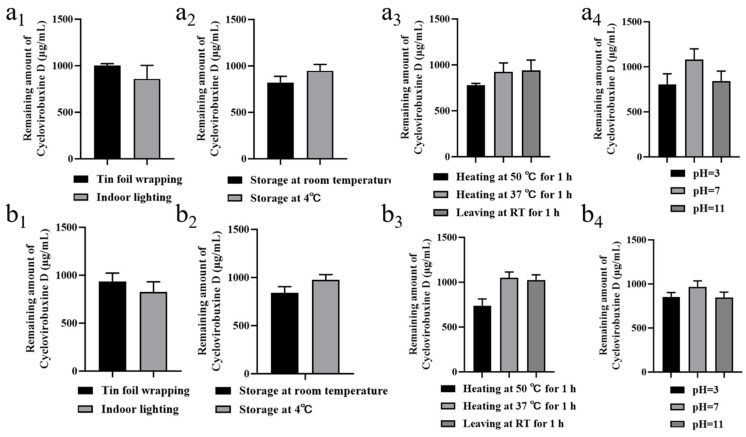
(**a_1_**) photostability; (**a_2_**) storage stability; (**a_3_**) thermal stability; and (**a_4_**) acid-base stability of 1 mg/mL cyclovirobuxine D solution. (**b_1_**) Photostability; (**b_2_**) storage stability; (**b_3_**) thermal stability; and (**b_4_**) acid-base stability of 1 mg/mL Cy-Nio. Among these, the statistical time for photostability and storage stability was 10 d, and the statistical time for thermal stability and acid-base stability was 1 h.

**Figure 6:**
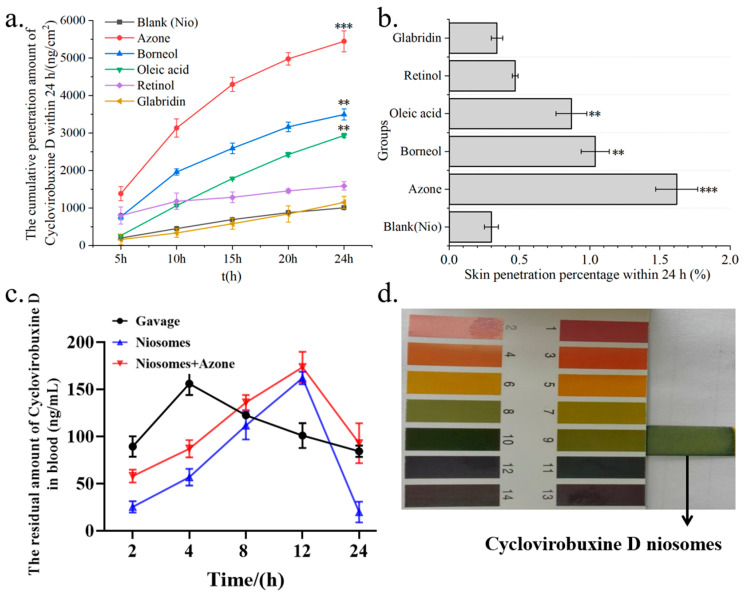
(**a**) Cumulative skin penetration; and (**b**) skin penetration percentage (%) of Cy-Nio under the condition of V (ethanol: saline) = 3:7 as the transdermal receiving liquid for different chemical penetration enhancers. (**c**) Pharmacokinetic curves of cyclovirobuxine D at different time points in the oral group, skin application group, and combination group of cyclovirobuxine D niosomes and azone. (**d**) The pH of cyclovirobuxine D niosome was detected using pH test strips. ** *p* < 0.01, and *** *p* < 0.001.

Furthermore, we conducted a thorough investigation of the stability characteristics of cyclovirobuxine D and Cy-Nio, evaluating their photostability, storage stability, thermal stability, and acid-base stability. Our findings revealed that the particle size of Cy-Nio remained relatively consistent under various storage conditions, including darkness, light exposure, and temperatures of 4 °C and room temperature, over durations of 1, 5, 10, and 15 days (Figure 4f,g). However, after 10 days of exposure to light at room temperature, we observed a decrease in the content of cyclovirobuxine D (Figure 5a_1_,b_1_). During heat treatment, we noted a reduction in the particle size of the niosomes after 1 h at 50 °C (Figure 4h), accompanied by a decrease in the content of cyclovirobuxine D (Figure 5a_3_,b_3_). Similarly, at 37 °C and at room temperature, the content of cyclovirobuxine D decreased after 1 h of treatment (Figure 5a_3_,b_3_), while the particle size remained essentially unchanged, suggesting that the niosomes can be stored at room temperature without alterations to their shape (Figure 4g). However, given the degradation of cyclovirobuxine D at room temperature (Figure 5a_2_,b_2_), we recommend storing it at 4 °C. Under acidic conditions at a pH of 3, we observed a decrease in both the particle size of the niosomes (Figure 4i) and the content of cyclovirobuxine D (Figure 5a_4_,b_4_). Notably, as the vesicles possess an alkaline pH range of 8–10 (Figure 6d), they were able to maintain their original particle size and cyclovirobuxine D content at a pH of 11 (Figure 5a_4_,b_4_), indicating the robustness and versatility of this niosome system.

### 3.5. Screening for Combined Administration of Cyclovirobuxine D Niosomes

The total dosage of cyclovirobuxine D administered was 1 mg (100 μL, 10 mg/mL). Upon analysis using LC–MS, the actual amount of cyclovirobuxine D encapsulated within the niosomes (Cy-Nio) was found to be 335,700 nanograms, or 0.34 mg. Based on the calculation formula, the encapsulation efficiency of Cy-Nio was determined to be 34%. For the transdermal delivery studies, a receiving liquid with a volume ratio of V (ethanol: saline) of 3:7 was selected. It was observed that Cy-Nio (0.30%) exhibited a skin permeation enhancement effect that was 1.5 times greater than that of the cyclovirobuxine D solution (0.20%). When azone was used as the chemical penetration enhancer, the skin penetration percentage of Cy-Nio reached a peak of 1.62%. Compared to Cy-Nio without any chemical penetration enhancers, the penetration enhancement ratio of azone was 5.43, followed by borneol (3.50), oleic acid (2.90), retinol (1.60), and glabridin (1.13) (Figure 6a,b). Furthermore, when compared to the cyclovirobuxine D solution at 0.20%, the penetration enhancement ratio of azone was 8.10 (1.62%/0.20%). Therefore, the combination of the chemical permeation enhancer azone with the niosomes of cyclovirobuxine D was chosen as our optimal formulation.

### 3.6. Transdermal Delivery of Cyclovirobuxine D Niosomes In Vivo

**Figure 7:**
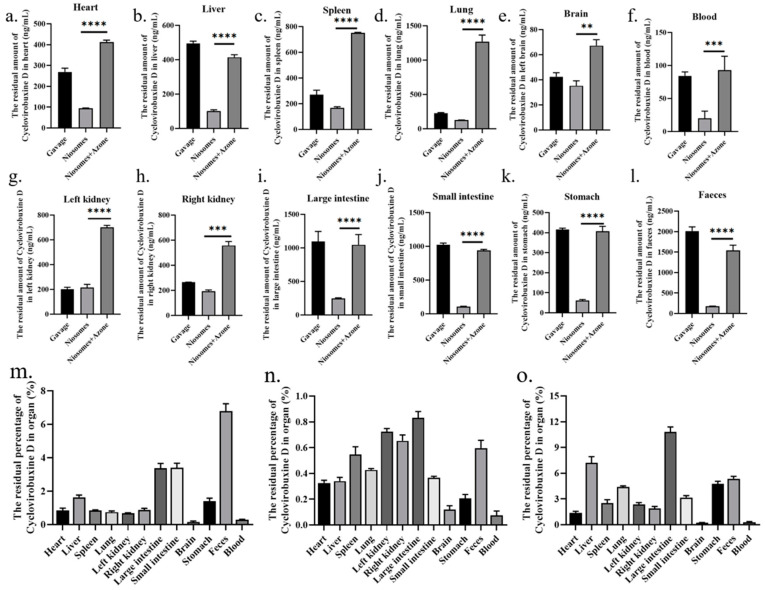
At 24 h after treatment with cyclovirobuxine D and Cy-Nio, the residual amount of cyclovirobuxine D in various organs of mice was detected, including: (**a**) heart; (**b**) liver; (**c**) spleen; (**d**) lungs; (**e**) brain; (**f**) blood; (**g**) left kidney; (**h**) right kidney; (**i**) large intestine; (**j**) small intestine; (**k**) stomach; (**l**) feces; and (**m**) oral method. (**n**) Cy-Nio; and (**o**) Cy-Nio were combined with azone to determine the percentage of residual cyclovirobuxine D in each organ. ** *p* < 0.01, *** *p* < 0.001, and **** *p* < 0.0001.

We selected the following three methods of administration for cyclovirobuxine D: oral ingestion; skin application using Cy-Nio; and combined skin application of Cy-Nio with the chemical penetration enhancer azone, each employing a dosage of 3 mg (300 μL, 10 mg/mL). Our findings revealed that, in contrast to oral ingestion, the transdermal delivery efficiency of Cy-Nio exhibited a reduction, which was evident in the decreased levels of cyclovirobuxine D in the heart, liver, spleen, lungs, kidneys, large intestine, small intestine, brain, stomach, feces, and blood after a 24 h post-administration dissection (Figure 7). Notably, the concentration of cyclovirobuxine D in the digestive organs, specifically the large intestine, small intestine, and stomach, decreased significantly (Figure 7i–k). This underscores the attenuation characteristic of transdermal delivery compared to oral ingestion. Within the initial 0–4 h, the blood levels of cyclovirobuxine D were higher in the orally administered group than in the skin-administered group. However, after 8 h, blood levels in the skin-administered group surpassed those in the orally administered group, suggesting that Cy-Nio possesses sustained-release properties, thereby prolonging the duration of cyclovirobuxine D’s efficacy (Figure 6c). Additionally, the incorporation of the chemical permeation enhancer azone into Cy-Nio significantly enhanced the transdermal delivery efficiency of cyclovirobuxine D, achieving a 1.88-10.31-fold increase compared to the untreated group (Figure 7). Interestingly, Cy-Nio alone revealed a higher residual amount of cyclovirobuxine D in the brain and heart compared to Cy-Nio combined with azone, which was 1.62 times and 1.48 times, respectively. This suggests that, under the influence of azone, it was more beneficial for cyclovirobuxine D to target the brain and heart. However, skin penetration enhancers typically do not act directly on the brain and heart. Instead, if the drug is able to be absorbed through the skin or other routes, and reaches adequate concentrations, it may indirectly affect brain and heart function. Given that cyclovirobuxine D plays a crucial role in the treatment of cardiovascular diseases, the regulation of blood pressure, and the protection of neurons in the brain, this targeted penetration enhancement holds great potential for the treatment of these diseases. Thus, we have demonstrated that the combination of niosomes and azone exhibits excellent permeation enhancement efficiency both in vitro and in vivo. Furthermore, due to the blood–brain barrier (BBB) effect, the amount of cyclovirobuxine D that crosses into the brain is minimal, representing the lowest concentration among all organs and failing to reach the upper limit of cyclovirobuxine D (Figure 7e).

### 3.7. Safety Assessment

**Figure 8:**
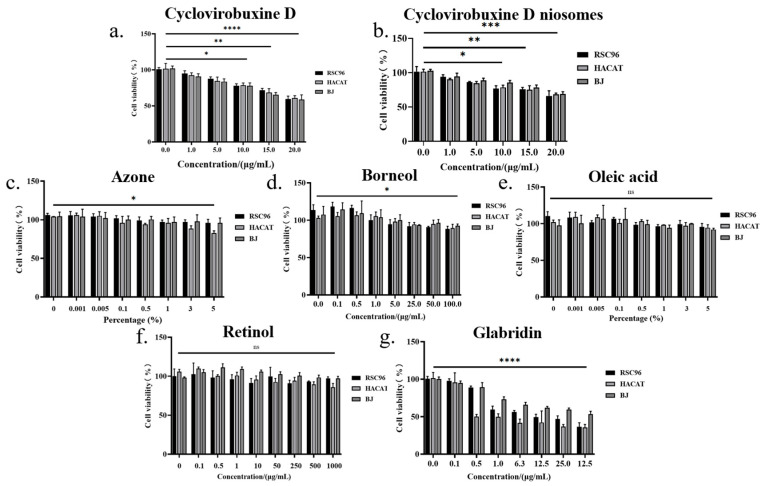
The effects of different concentrations of: (**a**) cyclovirobuxine D; (**b**) Cy-Nio; (**c**) azonel (**d**) borneol; (**e**) oleic acid; (**f**) retinol; and (**g**) glabridin on cell proliferation of RSC96, HACAT, and BJ cells. ^ns^ > 0.05, * *p* < 0.05, ** *p* < 0.01, *** *p* < 0.001, and **** *p* < 0.0001.

Research has shown that cyclovirobuxine D, in minimal concentrations, exhibits no inhibitory influence on the proliferation of three distinct cell types. However, at a concentration of 10 μg/mL, it demonstrates statistically significant (*p* < 0.05) toxicity towards RSC96, HACAT, and BJ cells. Furthermore, at a concentration of 20 μg/mL, its toxicity becomes highly statistically significant (*p* < 0.0001) (Figure 8b). Intriguingly, the formulation of niosomes mitigates the toxicity of cyclovirobuxine D, as depicted in Figure 8b. Moreover, the chemical permeation enhancers retinol and oleic acid exhibit virtually non-toxic profiles, as depicted in Figure 8e and 8f, respectively. Conversely, azone and borneol exhibit mild toxicity at elevated doses, with statistical significance (*p* < 0.05), as presented in Figure 8c,d. Nevertheless, glabridin poses a more significant toxic threat to these three cell types, as shown in Figure 8g.

**Figure 9:**
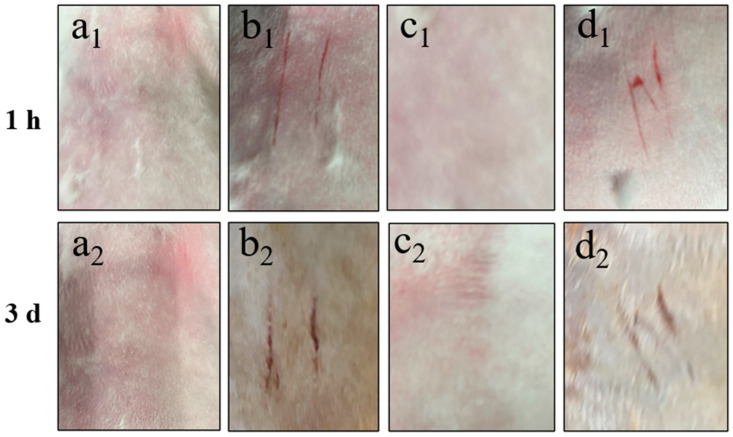
Skin irritation intensity of intact and scratched back skin of New Zealand white rabbit treated with Cy-Nio (contain azone) for 1 h and 3 d. Among them, (**a_1_**,**a_2_**) are the intact blank group skin, (**b_1_**,**b_1_**) are the scratched blank group skin, (**c_1_**,**c_2_**) are the intact Cy-Nio group skin, and (**d_1_**,**d_2_**) are the scratched Cy-Nio group skin.

In the skin irritation experiments, upon visual inspection three days post treatment, neither the intact skin group nor the scratched skin group of New Zealand white rabbits administered with Cy-Nio (containing azone) exhibited any signs of pigmentation, bleeding points, or alterations in skin texture, such as roughness or thinning, at the application site. Throughout both the administration and recovery observation periods, no irritant symptoms, such as erythema or edema, were observed at the site of application, as depicted in Figure 9. The skin irritation score for the scratched skin group was recorded as 0.30 ± 0.22, indicating a mild skin irritation accompanied by slight redness. Notably, there was no statistically significant difference (*p* > 0.05) in the skin irritation score of the control group, as shown in Table 5.

## 4. Discussion

The high incidence and mortality rates of cardiovascular and cerebrovascular diseases continue to be a major concern [4]. Cyclovirobuxine D has shown considerable promise; however, its physicochemical properties not only hinder its effectiveness but also pose potential toxicity risks. Given the necessity for lifelong medication in the elderly and the importance of convenient administration, the development of efficient and safe formulations is imperative and urgently needed [25]. Transdermal administration offers distinct advantages, including lower toxicity, precise dosage control, sustained effects, and ease of application. The analysis of samples using LC–MS after skin penetration revealed that the highest skin penetration of cyclovirobuxine D was observed in the 24 h dispersed solvent when the receiving liquid consisted of a ratio of V (PEG400: ethanol: saline) of 1:3:6, with a skin penetration percentage of 0.28% (Figure 2c,d). This underscores the difficulty in achieving therapeutic concentrations through skin administration alone. To overcome this challenge, our study evaluated the impact of five chemical penetration enhancers on the skin permeability of cyclovirobuxine D for the first time. We selected three Chinese standard chemical penetration enhancers, along with two additional compounds, to assess their influence on the skin penetration of cyclovirobuxine D. These chemical penetration enhancers were administered in combination with cyclovirobuxine D to achieve an appropriate dosage. When using a ratio of V (ethanol: saline) of 3:7 as the transdermal receiving liquid, azone emerged as the most effective enhancer for the cyclovirobuxine D solution, achieving a skin penetration-promoting effect of 4.55, while other chemical penetration enhancers demonstrated skin penetration-promoting effects ranging from 1.17 to 2.40 for cyclovirobuxine D (Figure 3a,b).

Traditional local dosage forms administer relatively large quantities of medication but can often elicit allergic reactions and are troubled by issues such as greasiness, staining, and toxicity. The emergence of transdermal nanocarriers has alleviated some of these challenges, enabling enhanced therapeutic effects through sustained release and minimal-to-no systemic toxicity [26]. Among these, niosomes represent novel vesicle systems that surpass conventional liposomes in terms of skin permeability. These vesicles, composed of Span60, ethanol, and cholesterol, possess optimal skin permeability characteristics. Their primary advantage lies in their ability to augment drug penetration into the skin while minimizing systemic absorption, thereby limiting the toxicity of various drugs to the skin layer [27]. Furthermore, Wei et al. crafted a cyclovirobuxine D (CVB-D) formulation based on chitosan nanoparticles (CS-CVB-D-NPs), which exhibited a suitable shape and size [28]. These nanoparticles featured continuous-release properties and were evaluated for the feasibility of delivering CVB-D to the brain via the nasal route. Consequently, a suitable nasal drug-delivery system was devised to enhance brain targeting [29]. Additionally, CVB-D encapsulated in angiopep-conjugated polysorbate 80-coated liposomes demonstrated superior brain targeting through intranasal administration [30]. This approach facilitates the localization of targeted drug-delivery systems within tissues, thereby modifying the delivery of therapeutic agents and formulations to their intended targets [31]. In this study, niosomes were prepared to encapsulate cyclovirobuxine D (Figure 4c), and their potential to improve skin penetration was explored. The results indicated that the niosomes increased skin penetration by 1.5 times (0.30%/0.20%) compared to the cyclovirobuxine D-dispersed solvent group. Notably, azone demonstrated the most potent promoting effect on niosomes, achieving a ratio of 5.43 (1.62%/0.30%) compared to Cy-Nio without chemical penetration enhancers (Figure 6b). Other chemical penetration enhancers exhibited skin penetration-promoting effects ranging from 1.13 to 3.50 on Cy-Nio. When the penetration enhancer azone was combined with niosomes, the penetration-promotion effect was 8.1 times (1.62%/0.20%) that of the CVB-D-dispersed solvent group.

Therefore, we chose to utilize the combination of Cy-Nio and azone to assess its skin permeability in animal models. Despite the considerable promise of transdermal drug-delivery methods, the barrier function of the stratum corneum restricts the number of drugs suitable for this route. Consequently, many drugs fail to achieve therapeutic concentrations through the skin following transdermal administration [32]. The key to overcoming this challenge lies in enhancing drug transdermal permeability and prolonging retention times, necessitating the development of specific dosage forms for administration. Our findings revealed that niosomes exhibited superior sustained-release properties compared to the oral administration group. Although the initial concentration of cyclovirobuxine D entering the bloodstream was lower than that of the oral group within the first 4 h, it demonstrated robust promotion of cyclovirobuxine D entry into the bloodstream between 8 and 24 h (Figure 6c), subsequently diffusing into various organs throughout the body. Furthermore, the incorporation of the chemical permeation enhancer azone augmented the transdermal delivery of cyclovirobuxine D, resulting in a residual enhancement ratio of various organs ranging from 1.88 to 10.31 times (Figure 7). The clinical application of cyclovirobuxine D is significantly hindered by its poor solubility, low absorption bioavailability, and severe toxic side effects. Addressing these limitations through formulation modification of cyclovirobuxine D is crucial. By controlling drug release through the skin, we can enhance patient compliance and dosage effectiveness [25], paving the way for broader clinical utilization.

In summary, this study introduces an innovative delivery system—a combination formulation of azone, niosomes, and cyclovirobuxine D. This system boasts a straightforward dosage form and convenient preparation process, significantly enhancing the solubility of cyclovirobuxine D, mitigating drug toxicity in vivo, and enhancing its circulation time. Consequently, it greatly improves drug bioavailability, laying the groundwork for a transdermal delivery strategy for cyclovirobuxine D and effectively addressing its clinical challenges. However, to date, no cyclovirobuxine D formulation has advanced to clinical trials. Bridging the gap from basic research to clinical trials necessitates in-depth studies on the formulation modification of cyclovirobuxine D. Therefore, exploring targeted formulation modification of cyclovirobuxine D and constructing a transdermal delivery system for it holds immense clinical significance, aiming to enhance therapeutic efficacy and minimize adverse reactions. Future research endeavors could also involve developing diverse formulations of cyclovirobuxine D, including liposomes, exosomes, and other vascular nanocarriers, which, despite offering advantages such as enhanced drug-delivery efficacy, improved pharmacokinetics, reduced toxicity, and better solubility and bioavailability for poorly water-soluble drugs, lack the ability to target specific organs or tissues for treating certain diseases. To overcome this limitation, we propose grafting various targeting peptides onto the surface of these niosomes, enabling them to carry cyclovirobuxine D and deliver it to the intended organs for superior therapeutic outcomes. These preclinical findings can be leveraged to design clinical studies on the developed topical niosomes of cyclovirobuxine D. Furthermore, these methodologies have already demonstrated success in treating diseases, such as cancer, with other drugs [33], suggesting their potential to revolutionize the therapeutic landscape of cyclovirobuxine D and beyond.

## 5. Conclusions

The employment of niosomes, in conjunction with the chemical penetration enhancer azone, for the transdermal delivery of cyclovirobuxine D represents a promising approach for skin-mediated systemic administration. Boasting a penetration enhancement ratio (ER) of 8.1, and exhibiting a sustained-release effect, this formulation holds considerable promise for clinical applications, promising high efficacy and safety in disease therapy. Both in vivo and in vitro experiments conducted on rats have validated its effectiveness, reinforcing its potential for translation into clinical practice.

## Figures and Tables

**Table 1:** Gradient elution procedure for cyclovirobuxine D chromatographic conditions.

Time (min)	Flow Rate (mL/min)	A%	B%
0–1min	0.35	90%	10%
1–3min	0.35	90–50%	10–50%
3–3.5min	0.35	2–50%	50–98%
3.5–4.5min	0.35	2%	98%
4.5–5min	0.35	90–2%	98–10%
5–7.5min	0.35	90%	10%

A% is the percentage of mobile phase A, B% is the percentage of mobile phase B.

**Table 2:** MRM ion pair parameters for cyclovirobuxine D mass spectrometry conditions.

Q1 (*m*/*z*)	Q3 (*m*/*z*)	DP (V)	CE (V)	EP (V)	CXP (V)
403.4	372.3	198	34	10	20
403.4	354.1	198	38	10	20

**Table 3:** Skin irritation response score.

Stimulus Response	Integral
Erythema	
No erythema	0
Mild erythema (barely visible)	1
Moderate erythema (visibly visible)	2
Severe erythema	3
Purplish-red erythema to slight eschar formation	4
Dropsy	
No edema	0
Mild edema (barely visible)	1
Moderate edema (significant bulge)	2
Severe edema (skin bulge of about 1 mm with a well-defined contour)	3
Severe edema (skin bulge of about 1 mm or more and enlargement)	4
The maximum total score	8

**Table 4:** Permeability-related indicators of all tested penetration enhancers.

	24 h Cumulative Penetration/(ng·cm^−2^)	24 h Cumulative Percentage/(%)	Enhancement Ratio (ER)	Flux/(J, ng/cm^2^/h)	Permeability Coefficient/(cm·s^−1^)
Blank	2954.40	0.10%	1	123.10	3.71 × 10^−4^
Retinol	3467.00	0.12%	1.17	144.46	4.31 × 10^−4^
Glabridin	5239.90	0.17%	1.77	218.33	6.62 × 10^−4^
Borneol	7091.90	0.24%	2.40	295.50	8.95 × 10^−4^
Azone	13470.40	0.45%	4.55	561.27	1.70 × 10^−3^
Oleic acid	4204.7	0.14%	1.42	175.20	5.31 × 10^−4^

**Table 5:** Skin irritation intensity score.

Group	Area	Skin Irritation Response Score After the End of Dosing		
		1d	2d	3d
Complete skin group	Administration side	0	0	0
	Control side	0	0	0
Scratch skin group	Administration side	0.40 ± 0.12	0.35 ± 0.20	0.30 ± 0.22
	Control side	0.36 ± 0.18	0.32 ± 0.25	0.26 ± 0.13

## Data Availability

All data and materials in this article are available.

## Supplementary Materials

The following supporting information can be downloaded at: https://www.mdpi.com/article/10.3390/pharmaceutics16121600/s1, Figure S1: The particle size meter repeatedly detects the particle size distribution range of niosomes.
