# Supplementary material for: Unlocking the Stratum Corneum Barrier to Skin Penetration for the Transdermal Delivery of Cyclovirobuxine D
Source: Pharmaceutics. 2024 Dec 16;16(12):1600. doi: 10.3390/pharmaceutics16121600 (PMC11678883; doi:10.3390/pharmaceutics16121600)
Supplement: Supplementary file 1 [file pharmaceutics-16-01600-s001.zip › pharmaceutics-3334982-supplementary.pdf]

pharmaceutics
MDPI

Supplementary Materials: Unlocking the Stratum Corneum Barrier to Skin Penetration for the Transdermal Delivery of Cyclovirobuxine D

Yun-Hao Ren, Feng-Yuan Song, Jing-Yu Zhao, Bing-Wen Liang and Li-Hua Peng

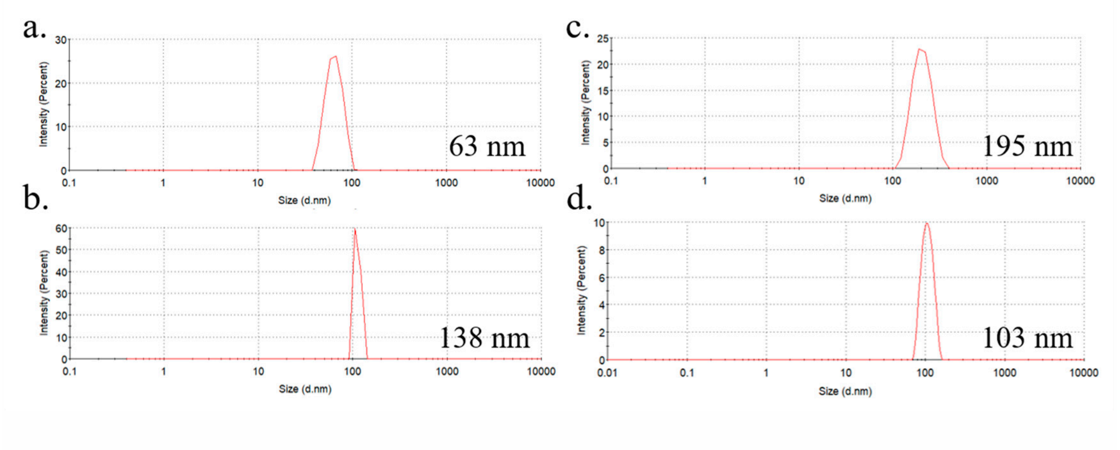

Figure S1. The particle size meter repeatedly detects the particle size distribution range of niosomes.
